# Practical application of genomic selection in a doubled-haploid winter wheat breeding program

**DOI:** 10.1007/s11032-017-0715-8

**Published:** 2017-09-03

**Authors:** Jiayin Song, Brett F. Carver, Carol Powers, Liuling Yan, Jaroslav Klápště, Yousry A. El-Kassaby, Charles Chen

**Affiliations:** 1 Forest and Conservation Sciences, Faculty of Forestry, The University of British Columbia, 2424 Main Mall, Vancouver, BC V6T 1Z4 Canada; 2 Department of Plant and Soil Science, Oklahoma State University, 371 Agriculture Hall, Stillwater, OK 74078 USA; 3 Department of Genetics and Physiology of Forest Trees, Faculty of Forestry and Wood Sciences, Czech University of Life Sciences, Kamycka 129, 165 21 Prague 6, Czech Republic; 4 Present Address: Scion (New Zealand Forest Research Institute Ltd.), 49 Sala Street, Whakarewarewa, Rotorua, 3046 New Zealand; 5 Department of Biochemistry and Molecular Biology, Oklahoma State University, Stillwater, OK 74078 USA; 6 Department of Biochemistry and Molecular Biology, 246 Noble Research Center, Stillwater, OK 74078 USA

**Keywords:** Genomic selection, Wheat, Single nucleotide polymorphism, Genomic best linear unbiased prediction, Reproducing kernel Hilbert space regression

## Abstract

Crop improvement is a long-term, expensive institutional endeavor. Genomic selection (GS), which uses single nucleotide polymorphism (SNP) information to estimate genomic breeding values, has proven efficient to increasing genetic gain by accelerating the breeding process in animal breeding programs. As for crop improvement, with few exceptions, GS applicability remains in the evaluation of algorithm performance. In this study, we examined factors related to GS applicability in line development stage for grain yield using a hard red winter wheat (*Triticum aestivum* L.) doubled-haploid population. The performance of GS was evaluated in two consecutive years to predict grain yield. In general, the semi-parametric reproducing kernel Hilbert space prediction algorithm outperformed parametric genomic best linear unbiased prediction. For both parametric and semi-parametric algorithms, an upward bias in predictability was apparent in within-year cross-validation, suggesting the prerequisite of cross-year validation for a more reliable prediction. Adjusting the training population’s phenotype for genotype by environment effect had a positive impact on GS model’s predictive ability. Possibly due to marker redundancy, a selected subset of SNPs at an absolute pairwise correlation coefficient threshold value of 0.4 produced comparable results and reduced the computational burden of considering the full SNP set. Finally, in the context of an ongoing breeding and selection effort, the present study has provided a measure of confidence based on the deviation of line selection from GS results, supporting the implementation of GS in wheat variety development.

**Electronic supplementary material:**

The online version of this article (doi:10.1007/s11032-017-0715-8) contains supplementary material, which is available to authorized users.

## Introduction

As the most globally planted cereal crop, wheat is one of the world’s most important food and protein source and among the top-traded agricultural commodities internationally (United States Department of Agriculture, 2016a, b). In order to sustain the vitality of such an important species, it is pivotal to improve the rate of genetic gain for wheat grain yield, with no compromise in market-ready end-use quality (Graybosch et al. 2014). With traditional breeding technology, a wheat breeding program would require about 7 or 12 years for spring or winter wheat, respectively, before a new variety is ready for commercial release (Baenziger and Depauw 2009). As the demand for wheat consumption exceeds current supply (United States Department of Agriculture 2016b), it is also imperative to incorporate emerging technologies into wheat breeding programs to ensure productivity meets these challenges.

Genomic selection (GS), which employs single nucleotide polymorphism (SNP) markers across the entire genome to predict an individual’s performance (Meuwissen et al. 2001), is a proven method to optimize and potentially accelerate the breeding process when centered on grain yield improvement; however, its return of investment could even be greater for the traits that are difficult or expensive to measure (Calus and Veerkamp 2011). Additionally, GS has the ability to substantially increase the selection intensity, thus providing scenarios for capturing greater gain per unit time. GS advantages have inspired scientists across the field of agriculture. For example, Bernardo and Yu (2007) carried out a simulation study demonstrating the advantage of GS in comparison to marker-assisted selection in maize; de los Campos et al. (2010) were the first to incorporate GS in wheat breeding by confirming that the inclusion of SNP markers resulted in improvement of GS model’s performance in predicting average grain yield. Since then, GS has gained increased acceptance in wheat breeding. Initial applications were focused on the exploration of only additive genetic variation among individuals, until later when de los Campos et al. (2009) extended the work of Gianola and van Kaam (2008) and utilized the reproducing kernel Hilbert space (RKHS) method to account for epistatic effects in addition to the additive effect and evaluated the method’s potential in wheat line evaluation. Subsequently, studies comparing the genomic best linear unbiased prediction (GBLUP) and the RKHS models’ performance were conducted (Crossa et al. 2010, 2011; He et al. 2016; Huang et al. 2016), without conclusive advantage to either approach.

While traditional phenotypic selection is considered time-, resource-, and space-consuming, in theory, adopting GS would create a paradigm shift where the genomic estimated breeding values (GEBVs) may be used to accelerate the breeding through early identification of parents or reinforce selection decisions made solely by phenotype-dependent field assessment. As a result, not only is genetic gain increased by rapid turnover of breeding cycles, but also effort and investment associated with breeding program development are substantially reduced (Baenziger and Depauw 2009). In addition, GS would enhance the utilization of multi-environment yield trials by considering the genotype by environment (G × E) effects in the prediction models, thus providing better selection decisions for best performers across several environments (Bassi et al. 2016) or for supporting farmers in realizing maximum yield potential through determining the best geographic positioning of candidate cultivars.

It has been a decade since the first study of GS in plant breeding was published (Bernardo and Yu 2007). Substantial evidence has shown the potential of GS in wheat breeding, with particular focus on increasing models’ predictive accuracies. However, very few studies have considered the practical implications of these results in the context of an active wheat breeding program. Thus, to elevate GS’s practicality, the objectives of the current study aimed to (1) address GS algorithm performance in predicting wheat grain yield across breeding cycles (breeding cycles were represented by years due to the uniformity of genotypes across years) in the line development stage, (2) investigate the possible upward bias in predictive ability from within-year cross-validation compared to cross-year, (3) explore the effect of considering G × E effect in the prediction models on their predictive abilities, and (4) evaluate the effect of SNP marker information on GS predictive ability.

## Materials and methods

### Phenotypic data

The efficiency of genomic selection across successive years was evaluated using a doubled-haploid (DH) population derived from a single cross between two commonly grown hard red winter wheat (*Triticum aestivum* L.) cultivars, ‘Duster’ (Edwards et al. 2012) and ‘Billings’ (Hunger et al. 2014). In total, 282 DH lines were developed, and among which, 257 lines were evaluated for grain yield in 2014 and 2015 at the Agronomy Research Station in Stillwater, OK, USA (36.12 N, 97.09 W). Plot size was 1.5 m × 3 m, with a sowing density about 200 seeds per m^2^, following a randomized complete block design. The soil type in Stillwater location was Kirkland silt loam or Norge loam (for details, see http://oaes.okstate.edu/frsu/agronomy-research-station/Stillwater_soilmap.pdf); in 2014, urea and diammonium phosphate (DAP) were applied (broadcast and incorporated) to achieve a final rate of 96 lb N/acre and 69 lb P/acre before planting. Similarly, in 2015, urea and DAP were applied to achieve a final rate of 91.5 lb N/acre and 57.5 lb P/acre. In both years, 239 out of the 257 lines were replicated twice, and the remaining 18 were screened for grain yield only once. When applicable, the mean of the two replications was taken to represent an individual line’s phenotype. Since the genotypes evaluated were identical for 2014 and 2015, the year effect was considered as environmental replications during data analysis. The 2014 trial was planted on 11 November 2013 and harvested on 20 June 2014. The 2015 trial was planted on 14 November 2014 and harvested on 14 June 2015. The total rainfall was 7.78 in. for the year 2014 growing season and 16.27 in. for year 2015. No trial received supplemental irrigation.

Days to heading (HD) were recorded for each DH line for both 2014 and 2015, as the variability of HD (measured as the duration from planting to heading) in wheat is an indicator of adaptability to its growing environment (Kiseleva et al. 2016). In addition, naturally occurring stripe rust (*Puccinia striiformis*) infection was observed in the 2015 trial. The infection type (severity) of stripe rust was recorded using the basic 0-to-9 scale described by Line et al. (1974), where 0 was completely resistant and 9 was completely susceptible; the incidence was rated by the modified Cobb USDA scale (Peterson et al. 1948); the severity of stripe rust was also recorded. Assessment of the rust infection was carried out on the 5th and 11th of May; at each time, the severity (RS_5_, RS_11_) and incidence (RI_5_, RI_11_) of the disease were recorded for each individual line.

### Genotypic data

Genotyping by sequencing (GBS; Elshire et al. 2011) was employed to generate genotypic data. The details of enzyme selection, library construction, and SNP data analysis can be found in Poland et al. (2012a) and Li et al. (2015). For the purpose of marker discovery, no reference information was involved in the SNP calling procedure; position information, such as chromosomes and positions, is not available for this SNP build. In total, 14,028 SNP markers were generated for these Duster × Billings DH lines, prior to other data treatment such as filtering and imputation.

To investigate the impact of missing data on GS models’ predictive ability, the SNP markers were grouped into five subsets based on the minimum call rate of 0.25, 0.4, 0.5, 0.6, and 0.75. Two imputation methods were employed to interpret missing data, namely mean imputation, which uses the average genotypic value of each SNP locus for all missing data, and the expectation maximization (EM) algorithm (Poland et al. 2012a).

### Statistical models

The performance of genomic prediction on grain yield of the 257 DH lines was evaluated using two different models: (1) GBLUP, a parametric model that accounts for only additive genetic effect, and (2) RKHS regression, a semi-parametric model that also considers correlations between markers, or non-additive effects.

Let *n* be the number of genotypes and *m* be the number of SNP markers. The GBLUP model takes the form

1 $$ \boldsymbol{y}=\boldsymbol{X}\boldsymbol{\beta } +\boldsymbol{g}+\boldsymbol{\varepsilon} $$

where ***y*** is the vector of phenotypic records of dimension *n* × 1, ***β*** is the vector of fixed effects containing the common intercept and other terms such as heading date and rust infection status, ***X*** is its corresponding design matrix, and ***g*** is the *n* × 1 vector of genomic breeding values, which were assumed to follow a normal distribution, $\boldsymbol{g}\sim N\left(0,\boldsymbol{G}{\sigma}_A^2\right)$, for which ${\sigma}_A^2$ is the additive genetic variance and ***G*** is the realized relationship matrix constructed following VanRaden (2008)

2 $$ \boldsymbol{G}=\frac{{\boldsymbol{ZZ}}^{\prime }}{2\sum {\boldsymbol{p}}_{\boldsymbol{i}}\left(1-{\boldsymbol{p}}_{\boldsymbol{i}}\right)} $$

where ***Z*** is the *n* × *m* matrix whose elements are defined as *a* − 2(*p*_*i*_ − 0.5), with *a* denoting the marker value as − 1 (homozygote), 0 (heterozygote), and 1 (alternative homozygote), and *p*_*i*_ is the frequency of the second allele at locus *i*; ***ε*** is the *n* × 1 vector of residuals with $\boldsymbol{\varepsilon} \sim N\left(0,\boldsymbol{I}{\sigma}_{\varepsilon}^2\right)$, where ***I*** denotes the identity matrix of order *n* and ${\sigma}_{\varepsilon}^2$ denotes the residual variance. The GBLUP model was implemented using the R package rrBLUP (Endelman 2011).

The semi-parametric RKHS model was carried out using a single Gaussian kernel. It is represented as

3 $$ \boldsymbol{y}=\boldsymbol{X}\boldsymbol{\beta } +\boldsymbol{u}+\boldsymbol{\varepsilon} $$

which has a similar form to GBLUP, but with a different assumption of $\boldsymbol{u}\sim N\left(0,\boldsymbol{K}{\sigma}_u^2\right)$, where ***K*** is the positive definite kernel matrix of dimension *n* × *n*, whose elements were the average squared-Euclidean distance between genotypes evaluated using the Gaussian kernel

4 $$ \boldsymbol{K}\left({\boldsymbol{x}}_{\boldsymbol{j}},{\boldsymbol{x}}_{\boldsymbol{k}}\right)=\exp \left[-h\times \frac{\sum_{l=1}^m{\left({x}_{jl}-{x}_{kl}\right)}^2}{m}\right] $$

where *x*_*jl*_ denotes the value of *l*th marker of individual *j* and *h* is the bandwidth parameter that determines the speed of decay of marker correlation as they get further apart in space. The RKHS model was implemented using the Bayesian approach in the R package BGLR (de los Campos et al. 2010).

The variables used to describe HD and rust infection were incorporated into the genomic prediction models using two methods: either directly as covariates with fixed effects or as correctors for the response variable, i.e., the phenotypes. The correction step was carried out using a simple linear model with the observed phenotypes as the response variable and one of the HD, RS, RI, or RS × RI as the explanatory variable. Residuals from the models were obtained to serve as the response variable in the genomic prediction models as *corrected phenotypes*. These two methods were implemented for fixed-effect variables in order to explore the differences in model behavior.

### Within- and cross-year prediction

Within-year cross-validation was performed for both 2014 and 2015 field evaluations separately. For each year, the data was randomly divided into ten folds, with nine folds as the training set and one fold as the validation set. Each run was repeated five times with different random folding. Evaluation of model’s predictive ability was based on the Pearson product-moment correlation between the GEBV and the observed phenotype (Obs) value (*r*_GS_ = *r*_GEBV , Obs_) of individuals in the validation set. First, only the random marker effect was included in the genomic prediction models and then HD were added into the models either as a fixed covariate or as a phenotype corrector. In 2015, RS_5_, RI_5_, RS_11_, RI_11_, and their products (RS_5_ × RI_5_ and RS_11_ × RI_11_) initially underwent a preselection, where each of the variables was introduced into the same prediction model with only random marker effect. These models were assessed based on their predictive abilities to choose the best variable to represent rust infection, which was later included in the cross-validation model in the same fashion as HD. For the purpose of simplifying this report, we only showed the best prediction model tested with the inclusion of rust infection covariable.

In the case of cross-year prediction, each of the 2-year data was treated as the training population, which followed the same scheme with ten folds and five replicates as the within-year cross-validation to obtain the GEBVs, to predict the performance in the other year. Since all genotypes remained the same between the field evaluations in 2 years, different years were considered as environmental replications, and the predictive ability was estimated by the Pearson product-moment correlation between the GEBV obtained from the training cycle and the observed phenotype value in the predicted breeding cycle.

For each scenario, both GBLUP and RKHS models were implemented for evaluation of their predictability. Model assessment was conducted across the combinations of five gradients of marker missing data ratio and two imputation methods. A grid search was also carried out for the bandwidth parameter *h* after the optimal composition of the RKHS model was acquired under each scheme.

### Prediction using G × E-corrected best linear unbiased prediction as phenotypes

In order to investigate the impact of incorporating G × E effect in the GS prediction models, we combined the 2-year data to fit a linear mixed-effects model using the R package lme4 (Bates et al. 2014) as follows:

5 $$ \boldsymbol{y}=\boldsymbol{X}\boldsymbol{\beta } +\boldsymbol{Zl}+\boldsymbol{Zi}+\boldsymbol{\varepsilon} $$

where ***β*** is the vector of fixed overall mean and year effects and ***l*** is the vector of random individual DH line effects following $\mathit{\operatorname{var}}\left(\boldsymbol{l}\right)\sim N\left(0,\boldsymbol{I}{\sigma}_l^2\right)$, where ${\sigma}_l^2$ is the individual DH line variance and ***i*** is the vector of random year × DH line interactions following $\mathit{\operatorname{var}}\left(\boldsymbol{i}\right)\sim N\left(0,\boldsymbol{I}{\sigma}_i^2\right)$, where ${\sigma}_i^2$ is the year × DH line interaction variance and ***Z*** is the incidence matrix assigning random effects to phenotypes in vector ***y***. The best linear unbiased predictions (BLUPs) of the random individual line effect were extracted to be used as the response variable in the GBLUP and RKHS models as the G × E-corrected phenotype to predict lines’ mean performance in each individual year. No other environmental variables were included in these GS models later on.

### Marker selection

To discuss the redundancy that might have been caused by the correlation and linkage of SNP markers, we examined the marker information most efficiently used for prediction. The evaluation of marker selection also followed a tenfold cross-validation scheme. Starting with the marker array at the SNP call rate determined by the models having the highest predictive ability from within-year cross-validation and cross-year prediction, a matrix of marker pairwise correlation was then calculated using the R package corpcor (Opgen-Rhein and Strimmer 2007; Schäfer and Strimmer 2005). The matrix construction, along with the succeeding steps, was carried out within each run using the marker information from the training population (TP) only. The set of markers was subsequently filtered by removing those with any absolute pairwise correlation higher than a threshold value, *t* (*t* = 1.0, 0.9, 0.8, 0.7, 0.6, 0.5, 0.4, 0.3, 0.2). The resulting SNPs were then used to construct the genomic relationship matrix for fitting the two best genomic prediction models in order to obtain predictive ability.

### Consistency of elite line selection

For both years, the individuals were ranked based on their GEBVs from the best-performing models. The comparisons were made to show differences in models that did and did not consider G × E effect. For models that did not consider G × E effect, there were four models within each year: the best cross-validation models using GBLUP and RKHS and the best cross-year prediction models using GBLUP and RKHS. For models that considered the G × E effect, two models within each year were evaluated: the best GBLUP and RKHS models trained with G × E-corrected phenotypes. Individual with the lowest GEBV was ranked the first, and individual with the highest GEBV was ranked the last, resulting to four rankings for non-G × E models and two rankings for G × E models for each DH line in every year. The mean of these indices was taken to represent each individual’s ranking within that year. The entire population was then sorted by their mean rank indices in year 2014 from the highest to the lowest, and the ranking distance (*d*) was calculated as the absolute difference between the individual’s mean rank index in 2014 and its corresponding mean rank index in 2015. Starting with the ranking distance of the highest-GEBV individual in 2014, by adding on the absolute difference of the next individual in the hierarchy (which is the second highest-GEBV individual), the average ranking distance ($\overline{d}$) was estimated by the mean of this total, and so on

6 $$ \overline {{\boldsymbol{d}}_{\boldsymbol{n}}}=\frac{\sum_1^{\boldsymbol{n}}{\boldsymbol{d}}_{\boldsymbol{n}}}{\boldsymbol{n}}\left(n=1,2,\dots, 257\right) $$

The final product was a vector of length 257, which allowed observing the change in the average ranking distance from the best-performing individual to the worst.

In order to achieve consistent selection of elite lines from this population, a relatively shorter distance for highly ranked individuals was expected, so that they are more likely to be chosen for further breeding regardless of the environmental differences.

## Results

### Data description

The average HD in 2014 were 170.37 (±1.93) days, slightly longer than that of 2015 (161.81 ± 1.13 days) (*p* < 0.0001). The severity and spread of rust infection both increased from the first to the second time point (Table S1). Grain yield in 2014 had a mean of 1579.73 (±266.31) kg ha^−1^, which was lower than that of 2015 (2228.03 ± 443.86 kg ha^−1^) (*p* < 0.0001). Grain yield in 2014 and 2015 had a positive correlation of 0.417. Before any data treatment, the overall SNP call rate averaged at around 0.6. Filtering for SNP’s call rate higher than 0.75, only 4010 SNPs remained; however, 12,944 SNPs can be obtained if considering a SNP call rate higher than 0.25 (Table S2).

### Genomic prediction model performance

#### Effects of missing genotype data and imputation methods

**Fig. 1:**
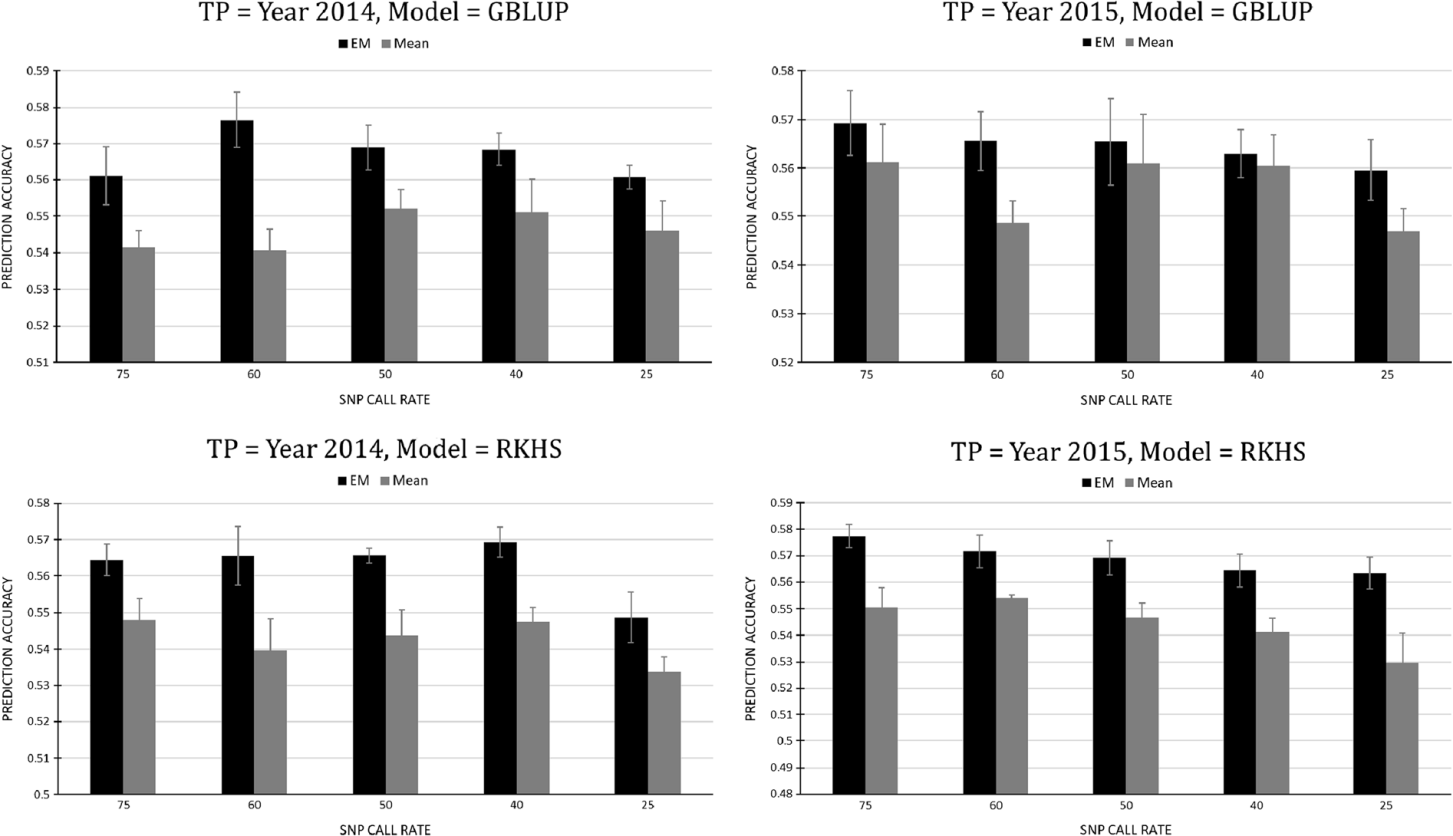
Comparison of two missing data imputation methods, EM and mean, based on the predictive ability from the GBLUP (above) and RKHS (below) cross-validation models (with SNP effect only) across a gradient of SNP call rate; TP training population; bandwidth parameter was set to 0.1 for all RKHS models

Two imputation methods, mean and EM, were evaluated by the within-year cross-validation models across a gradient of SNP call rate using 2014 and 2015 population, respectively (Fig. 1). In the evaluation of SNP call rate impact on predictive ability, only random marker effect was included in the tested models. In general, EM imputation outperformed the mean imputation in every scenario; effects of SNP call rate were little in 2015, as using the 4010 SNPs that have a call rate higher than 0.75 generated similar results with the rest of SNP call rate categories for both imputation methods. However, the effect of SNP call rate was more significant in 2014—the best predictability (prediction ability = 0.58) was observed when SNPs have the call rate of > 0.6, while the lowest prediction ability (0.54) was found in the SNP call rate of > 0.25 and imputed with the mean (for details, see Fig. 1). The difference of SNP call rate might seem small; however, our comparison between mean and EM imputation methods indicated that the latter consistently produced better model performance (Fig. 1). Poland et al. (2012a) found only lower imputation error from EM than population mean for the masked non-missing genotypes, with no advantage of EM imputation in predictive ability for yield. Possible reasons for this discrepancy could be due to the relatedness between the training and validation population. In Poland et al. (2012a)’s study, there were no common full sib lines between the training and validation sets, while the DH lines in our Duster × Billings population shared common parentage. Our results confirmed the superiority of EM over mean imputation for the DH populations where progeny is in moderate to high relatedness; due to its observable superiority for our populations, EM imputation was used for all the following analyses.

#### GS predictability evaluation

Given the considerable number of models tested in this study and for the purpose of simplifying their comparisons, models with the highest predictive abilities were chosen to represent the four tested model’s scenarios. In each scenario, the best-performing GBLUP and RKHS models were selected for each variable combination: SNP marker only, SNP marker + HD, SNP marker + rust rating, and SNP marker + HD + rust rating. The latter two combinations were only available to models that used 2015 as training population.

#### Within-year prediction using 2014 as training population

With only the realized relationship matrix ***G*** in the model, GBLUP and RKHS had similar performances when 2014’s grain yield was used as the training population for within-year cross-validation: both models yielded the highest predictive ability of ~ 0.57 with the SNP dataset that had a SNP call rate higher than 0.6 and was imputed with the EM algorithm (Table 1). When HD were included in the model either directly as a covariate or indirectly as a corrector for the phenotype, the predictive ability decreased for GBLUP but increased for RKHS. Both models required more SNPs to attain their highest predictive abilities (i.e., correlation between GEBV and Obs) with inclusion of HD (Table 1).

**Table 1:** Best-performing models and the number of SNPs required

Training population	Algorithm	Model	Prediction population			
			Predictive ability (± SE)		Number of SNPs (call rate)	
			2014	2015	2014	2015
2014	GBLUP	G	0.58 (±0.008)	0.35 (±0.002)	5726 (0.6)	7260 (0.5)
		G + HD	0.57 (±0.003)	0.33 (±0.002)	7260 (0.5)	5726 (0.6)
	RKHS	G	0.57 (±0.005)	0.36 (±0.003)	5726 (0.6)	7260 (0.5)
		G + HD	0.65 (±0.005)	0.42 (±0.003)	7260 (0.5)	5726 (0.6)
2015	GBLUP	G	0.37 (±0.003)	0.57 (±0.007)	9244 (0.4)	4010 (0.75)
		G + HD	0.38 (±0.002)	0.55 (±0.004)	7260 (0.5)	5726 (0.6)
		G + rust (RI_5_)	0.37 (±0.003)	0.56 (±0.006)	4010 (0.75)	5726 (0.6)
		G + HD + rust (RI_5_)	0.37 (±0.006)	0.53 (±0.006)	7260 (0.5)	4010 (0.75)
	RKHS	G	0.39 (±0.003)	0.60 (±0.004)	7260 (0.5)	4010 (0.75)
		G + HD	0.39 (±0.003)	0.61 (±0.003)	5726 (0.6)	5726 (0.6)
		G + rust (RI_5_)	0.40 (±0.001)	0.68 (±0.003)	7260 (0.5)	5726 (0.6)
		G + HD + rust (RI_5_)	0.39 (±0.002)	0.70 (±0.003)	7260 (0.5)	5726 (0.6)
BLUP	GBLUP	G	0.48 (±0.003)	0.52 (±0.006)	5726 (0.6)	5726 (0.6)
	RKHS	G	0.50 (±0.003)	0.56 (±0.006)	5726 (0.6)	7260 (0.5)

*SE* standard error

#### Within-year prediction using 2015 as training population

Among the models trained with 2015’s grain yield data, for within-year cross-validation with only marker effect, RKHS resulted in a higher predictive ability (0.60 ± 0.004) than GBLUP (0.57 ± 0.007). Similar with year 2014, the inclusion of HD did not show improvement in model performance for GBLUP, while a modest improvement for RKHS was observed when fitting HD as a covariate. In addition to HD variation, the impact of the rust infection on year 2015 prediction models was evaluated in the same fashion as HD. Both models showed better predictive ability performance with rust infection variables included compared to models that had only HD. GBLUP and RKHS achieved their highest predictive abilities when RS_11_ and RI_5_ were included as covariates, respectively. Further, fitting both HD and RI_5_ as covariates resulted in the best model performance for RKHS; interestingly, the GBLUP showed the worst model performance when including both HD and RS_11_ as covariates (Table 1).

#### Cross-year prediction using 2014 as training population

Trained with 2014 grain yield data to predict 2015’s grain yield resulted in approximately 41 and 36% reduction in predictive ability for GBLUP and RKHS models, respectively. Using linear GBLUP, models with only SNP marker data produced the best cross-year predictive ability (0.35 ± 0.002); inclusion of covariates showed a negative impact on the model performance (Table 1). On the contrary, RKHS prediction performed better when HD were included as a covariate, and this model, in fact, was the best predictive model among all cross-year predictions (0.42 ± 0.003). Overall, the predictive ability for cross-year prediction for 2014 yield data was at 0.36 (±0.025), significantly lower than that for within-year cross-validation (Table 1).

#### Cross-year prediction using 2015 as training population

In general, cross-year prediction results showed higher consistency when 2015 was used to predict 2014 (average predictive ability, 0.38 ± 0.014). Also, to predict grain yield in 2014, RKHS consistently performed better; even in cross-year prediction without covariates, RKHS outperformed the linear GBLUP model, as opposed to the slightly higher accuracy estimate obtained from GBLUP in the 2014 within-year cross-validation (0.58 and 0.57 for GBLUP and RKHS, respectively). The best performance was obtained when RI_5_ was included in the RKHS model as a covariate, though inclusion of both HD and rust ratings (RI_5_) produced comparable results (Table 1).

To summarize the predictive ability performance for the two consecutive years’ yield data, results from RKHS, in general, produced higher accuracy than that from GBLUP (Table 1). The RKHS also benefited considerably from the inclusion of covariates; GBLUP was at its best only when SNP markers were used, except for the very slight gain of predictive ability in the cross-year prediction when 2015’s data was used to predict 2014’s grain yield and HD was included as a covariate (0.37 versus 0.38). As shown in Table 1, the inclusion of covariates like HD and rust infection ratings was not recommended for GBLUP; in the case of within-year cross-validation using 2015 yield data, prediction performance for GBLUP was, in fact, at its lowest when covariates were included in the model (Table 1).

Also, predictive ability for within-year cross-validation ranged from 0.53 (GBLUP, TP = 2015, SNP marker data + HD + rust rating) to 0.70 (RKHS, TP = 2015, SNP marker data + HD + rust rating). Taking the year effect into consideration, predictive ability was dramatically reduced to 37%. This evident decrease in prediction accuracy might suggest an overestimation of predictability when the conventional cross-validation procedure is used to evaluate model performance.

To investigate the factors affecting model performance, we also compared the numbers of SNP markers and the missing data ratio to determine the genetic information content required for predictive analysis. Cross-year prediction models required a larger number of SNP markers for eight out of the 12 scenarios to achieve comparable prediction results, suggesting the complex genetic architecture of grain yield trait (Table 1). Finally, search for the optimal bandwidth parameter *h* across models failed to identify a single bandwidth value. The pattern changed with the training population, model composition, and the number of markers employed (Fig. S1, S2). According to our results, an *h* value between 0.1 and 1 is recommended for the acquisition of the highest predictive ability.

#### Impact of accounting for G × E effect on model predictability

Models trained with BLUP phenotypes showed consistent improvement on predictive abilities for both GBLUP and RKHS compared to the cross-year prediction models. Noticeable increases of 26 and 49% in predictability were observed for GBLUP when predicting grain yield in 2014 and 2015, respectively (Table 1). Similar improvement of model performance was also observed for the RKHS models (25% for predicting 2014 and 33% for predicting 2015, Table 1). In between predictive algorithms, RKHS again outperformed GBLUP in predicting grain yield, with an average of 6.0% increase of predictability observed in RKHS models.

#### Marker selection

The best model for within-year cross-validation was the RKHS with TP = 2015, SNP marker data + HD + rust rating, while the best model for cross-year prediction was also RKHS with TP = 2014, SNP marker data + HD, and both models required the marker subset at a call rate of 0.6; hence, 5726 SNPs were considered to be a reasonable starting point of our investigation on marker selection. Based on the whole population SNP marker data, the number of SNPs remained after filtration by the absolute pairwise correlation value *t* is shown in Table 2. Since the filtration step was carried out within each fold, the pairwise correlation value was estimated based on the training set rather than the whole population. The values in Table 2 could be seen as a reference, while the actual number of SNPs varied with the changing training populations.

**Table 2:** Number of SNP markers within each correlation group based on the whole population data

	Absolute pairwise correlation threshold (*t*)									
	Full set	1.0	0.9	0.8	0.7	0.6	0.5	0.4	0.3	0.2
No. of SNPs	5726	4976	4506	4241	3883	3338	2595	1473	267	27

**Fig. 2:**
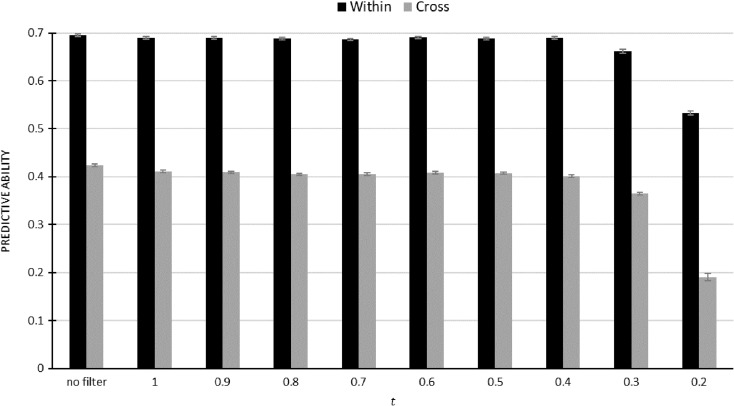
Predictive ability from the best within-year cross-validation model (within: year 2015 RKHS model with the marker effect and both heading date and disease index as covariates) and the best cross-year prediction model (cross: year 2014 predicting 2015 RKHS model with the marker effect and heading date as covariate) across subsets of marker filtered by absolute pairwise correlation threshold (*t*)

Overall, a comparable pattern of predictive ability was observed for both within-year and cross-year models (Fig. 2), where predictive ability remained constant until *t* = 0.4, and at *t* = 0.3, the prediction abilities from both within-year and cross-year models were reduced, showing a significant loss of information due to the sparse marker density. For the DH population, approximately 1500 SNPs at the absolute pairwise correlation threshold of *t* = 0.4 could result in a similar predictive ability when the full set of 5726 SNPs was used.

#### Consistency of elite line selection

**Fig. 3:**
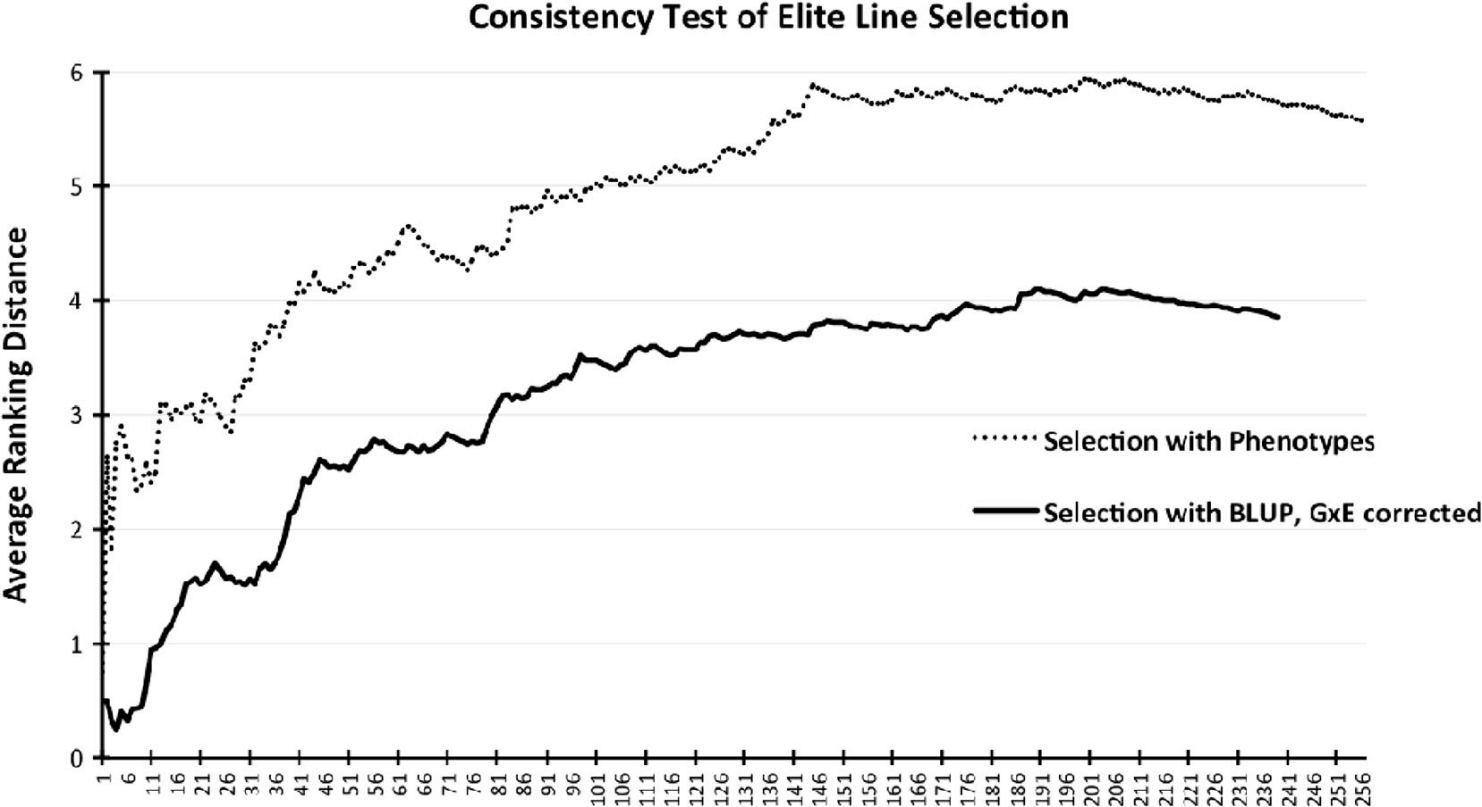
Trend of average ranking distance over the number of individuals from models which do and do not consider the G × E effect. Analysis started with the ranking distance of the best-performing individual in the year 2014 and proceeded by adding the next best individual’s ranking distance and taking the mean until all individuals in the population were included (18 individuals were removed from the G × E models due to the missing replicates)

For the models that did not consider G × E effect, the trend of average ranking distance ($\overline{d}$) has shown some substantial fluctuation among the top 30 lines, but the top 10% individuals produced consistent distance values under or close to 3, indicating that the selection of the best individuals was consistent across different environments (Fig. 3). The number then gradually increased and plateaued at an average distance close to 6 when about 80% of the population was included, suggesting that evaluating the majority of the population with moderate performances was less certain relative to the top individuals. There was also a slight decrease in the average distance with inclusion of the lowest-ranking individuals, showing a steady assessment for those poorly performing lines. A similar pattern could be observed for models that considered the G × E effect, but the average distance is, in general, 2 units smaller than the models without the G × E effect, indicating a higher consistency of selection when trained with G × E included. In summary, the models were more consistent in selecting individuals with extreme performances than evaluating average lines, and incorporation of the G × E effect into the model improved the selection consistency.

## Discussion

### Prediction model comparison

Efficacy of genomic selection has been widely explored since its inception in 2001 (Meuwissen et al. 2001). Evidences for GS’s potential in wheat breeding programs were demonstrated by a number of studies (e.g., Crossa et al. 2010, 2011; Poland et al., 2012a; He et al. 2016; Huang et al. 2016; Michel et al. 2016; Saint Pierre et al. 2016); few of these studies were focused on the prediction performance across breeding cycles or considered the application in actual breeding programs. In the present study, we assessed the predictive ability of genomic selection models using grain yield data from two successive years of a hard red winter wheat DH population as an example. Focusing on grain yield production, our results showed the superiority of RKHS over the linear additive alternative, GBLUP. This observation corresponds to a number of previous studies that investigated genomic selection model performance for grain yield prediction. For example, Huang et al. (2016) reported similar disparity in accuracy, ~ 33% difference between RKHS and GBLUP in predicting grain yield for 273 elite soft winter wheat lines. With a larger collection of 2325 European elite winter wheat lines, He et al. (2016) attained 5% higher predictive ability that was associated with 17% reduction in standard error for RKHS than GBLUP when evaluating grain yield in multiple sites. Additionally, RKHS outperformed other methods such as BayesCπ and artificial neural networks by 4% when predicting wheat grain yield (Heslot et al. 2012); RKHS also surpassed Bayesian LASSO (a similar algorithm to GBLUP but with marker-specific shrinkage) for within-year cross-validation in grain yield using 599 wheat lines and 94 elite spring wheat lines (Crossa et al. 2010, 2011). Also, our results suggest the advantage of RKHS’s broad applicability in predicting polygenic, complex traits like grain yield.

The strength of predictive algorithms diminished significantly (on average, a 0.22 decrease in predictive ability) from within-year cross-validation to a more realistic cross-year prediction; in the present study, this decrease in predictive ability was present in both parametric and non-parametric algorithms for all scenarios (Table 1), where, on average, a 36 and 38% decrease in predictive ability was observed, switching from within-year cross-validation to cross-year prediction for GBLUP and RKHS, respectively. Michel et al. (2016) also observed a major decline in the predictive ability for cross-year prediction in comparison to within-year cross-validation in a 5-year study for 659 commercial winter wheat lines. Similarly, an average accuracy reduction from 0.65 to 0.5 was reported by He et al. (2016); a much larger decrease of 50% in predictive ability was also reported in a two-generation sugar beet study (Hofheinz et al. 2012). Such inflation of predictive ability calls for caution when only evaluating GS applicability with within-year cross-validation, as this could be the result of the confounding effect of common environmental variation (Lorenz et al. 2011). In most of the literatures that estimate predictive ability based on cross-validations, the Pearson product-moment correlation between GEBV and true breeding value (TBV), *r*(GEBV, TBV), was used to reflect the confidence of how GEBV can be used to replace field evaluation. Since TBV is unknown, we could only measure the observed phenotype (Obs) and evaluation of model performance is based on *r*(GEBV, Obs), which is assumed to be the product between *r*(GEBV, TBV) and *r*(Obs, TBV). This assumption is only valid when the common element between GEBV and Obs is just TBV, and more importantly, the assumption of uncorrelated error terms between GEBV and Obs also needs to remain true. It can be expected that having both training and validation sets evaluated in the same environment in the same year constitutes a violation to the assumption of uncorrelated error terms; additionally, the presence of G × E is expected to produce an upward bias in predictive ability for within-year cross-validation. When evaluating the applicability of GS, the present study along with others also concludes that cross-year prediction should be considered.

The bandwidth parameter (*h*) in RKHS is used to control the rate of decay of the covariance between genotypes. For cross-year predictions of a single-cross DH population, in theory, a single value of *h* could be expected, given no new recombination events between genotypes. Our results, however, found an inconclusive result for the bandwidth parameter (Figs. S1, S2). The search for a single optimal bandwidth parameter was also discussed in the original work that proposed the use of RKHS for genomic selection, as de los Campos et al. (2010) indicated that a variation of the optimal value of *h* is expected when there is change in the distribution of observed genetic distances, which in part could be due to the different numbers of SNP markers used in our study. Other factors such as the genetic architecture of trait of interest and choice of kernel function also affect the estimate of this parameter (de los Campos et al. 2010). Cross-validation is commonly used as independent evaluation to identify the optimal value for the bandwidth (Härdle and Linton 1994), and alternatives like the kernel averaging method proposed by de los Campos et al. (2010) and Bayesian-based selection of *h* in Pérez-Elizalde et al. (2015) can also be considered without going through a large number of grid search.

The strength of RKHS in capturing additive and non-additive genetic effects, including high-level interaction terms, was recommended by Gianola and van Kaam (2008). This observation was largely agreed in our within year cross-validation results. However, as reported in a number of studies (for example in Heslot et al. 2012), the possibility of RKHS model’s overfitting issue could lead to a larger degree of variability. In *k*-fold cross-validation, the evaluation of model performance can be divided into bias and variance components. While unbiasedness is cited as the beneficial quality of a model, low variance is just as important. This notion of overfitting could be a more serious concern when GS is applied at the initiation, where a large number of crosses are made to screen for potential targets. At the line development stage of a winter wheat breeding program, our results show promising outcomes of RKHS in predictability for both means and variability in *k*-fold cross-validation, suggesting its superiority in capturing complex polygenic trait variation in a steady environment. In cross-year prediction, the gain of predictability of RKHS was still apparent, especially when using year 2014 as training population to predict year 2015. The best cross-year predictability was obtained from training on year 2014 to predict grain yield in 2015 (0.42 ± 0.003, Table 1), with heading date covariable to control environmental variability that is possibly due to drought (7.78 versus 16.27 in. for year 2014 versus 2015; Grogan et al. 2016; Kiseleva et al. 2016). Also supported by the model comparison (Table 1) to yield compatible predictability, the use of individuals evaluated in a relatively stable year as training information is recommended; this finding is in accordance with Saint Pierre et al. (2016), in which the highest predictive ability was from an environment without the presence of any dominant biotic or abiotic stresses.

In our case where annual rainfall in 2014 was significantly lower than 2015, considering phenotypes for the G × E effect in training population produced higher predictive abilities for both GS algorithms compared to the cross-year scenarios (Table 1). This observation accords with several previous studies that also consider the G × E effect: Burgueño et al. (2012) assessed multi-environmental effects using a multivariate GBLUP model and found it to be superior to its single-environment counterparts, prediction accuracy of the G × E model was significantly higher for complex traits like grain yield in Zhang et al. (2015), and Saint Pierre et al. (2016) observed the best model performance when the interaction between environment and genotype was included. Our results confirmed that adjustment of phenotypes for cross-year G × E effect has a positive impact on GS model’s predictability, which makes the selection of superior lines across environments possible (Fig. 3). The better performance of RKHS compared to GBLUP using G × E-corrected BLUP as phenotypes in the training population reinforced RKHS’s advantage over its linear counterpart and rendered it suitable for predicting complex traits like grain yield.

### Marker selection

The unprecedented efficiency of next-generation sequencing technology has created a paradigm shift that changes genetic research from trait-driven science to genetic-driven discovery. Accompanied with this rapid advancement, issues in data-information inequality have become increasingly important as *information volume* is often smaller than *data volume*. Simulation studies of dairy cattle and corn breeding showed that accuracies of prediction first increased with the number of SNPs and then plateaued in spite of the growing quantity of markers (Habier et al. 2013). In another study of a closely related wheat population, the authors postulated a comparable performance of 1827 selected SNP markers relative to 34,749 SNPs (Poland et al. 2012a). Similar predictability for wheat grain yield was achieved with variable genetic marker densities (see Crossa et al. 2010; de los Campos et al. 2009 versus Crossa et al. 2011). Using a cross-environment validation, our results indicated that approaching a comparable level of predictive ability in grain yield of a winter wheat single-cross DH population requires only a moderate number of SNP markers. Such a lack of improvement with additional data points (i.e., more SNPs) is not only the resource for inefficiency but also the underlying cause of correlated errors. The level of line development in the population and the relatedness among individuals are the two main contributors to reaching this plateau (de los Campos et al. 2013). With our single-cross DH population, the long spans of LD in the genome, and high relatedness within the training population and between training and validation populations, filtering the SNP markers based on their correlation coefficients could produce a satisfactory predictive ability while requiring less computational effort and time.

## Perspectives

Over the past decade, a substantial amount of efforts has been dedicated to exploring and evaluating the applicability of genomic selection for crop improvement. With a more realistic cross-year validation across highly diverse production environments, our results verified the superiority of the RKHS method over the whole-genome regression GBLUP. Further, model performance evaluation based on within-year cross-validation is likely to be biased, and when aiming to shorten the time required for line development or to optimize selection during line testing stage, a more ideal design like our 2-year validation should be considered with multilocation field data to handle correlated errors.

Up to this date, only a few attempts were made to investigate the realized outcome from GS in the context of an ongoing breeding program. Among these, our study demonstrated that greater confidence of line selection based on genomic selection could be achieved and line selection encompassing high breeding values with precision should be considered as a prerequisite, before further implementation (Blondel et al. 2015). Given the variability in predictive abilities among the various models examined in the present study, forward selection for high-performing lines was proven consistent with minimal ranking differential, even when a moderate number of SNP markers were used. Though the differential was slightly larger, rankings of low-performing lines were also considered stable. In summary, the robust assessment provided by our ranking distance measurement in line selection supports the advantage of genomic selection as a supplemental selection tool.

In traditional breeding wheat programs, once the main objectives are determined, for example to improve grain yield and adaptability, the breeding cycle is initiated with the hybridization stage. Following hybridization, efforts are made to reduce within population heterozygosity but maximize heterogeneity, while concurrently selecting desirable progenies for further assessment. Since wheat largely self-pollinates, the proportion of heterozygous loci decreases by 50% with each selfing generation. As the breeding program advances until reaching an acceptable level of homozygosity, a considerable amount of alleles contributing to the target traits could be lost due to drift. As a result, retaining a high level of desired alleles in the population is a difficult task, yet crucial for breeding practices. When the level of inbreeding reaches the desired level, a round of selection is made to create desirable lines. The main objective of such breeding and selection programs then becomes trait evaluation, including the physical characteristics of grain, and reaction to biotic and abiotic stresses, including resistance to a suite of diseases (which in the Great Plains may include ten or more diseases). Before elite lines reach commercial release, finalists must go through extensive evaluation in replicated yield trials. Due to highly correlated environmental effects within a specific year, 3 years of replicated yield evaluation is commonly practiced and this is considered the absolute minimum for variety release. More often, fixed lines will be subjected to five or more years of replicated yield and quality testing. Finally, after 7 to 12 years of hybridization, population development and inbreeding, and line development testing, often only a single cultivar is released from an initial pool of 50,000 to 60,000 lines in the intermediate inbreeding generations.

With the incorporation of recent advanced technologies, this long-term (7–12 years) endeavor could be significantly shortened. The development of DH lines could reach 100% homozygosity in a single generation compared to five to ten generations in the traditional method. The application of GS shown in this study, especially for those that adopt whole-genome regression approaches, is capable of increasing the precision of line selection provided by the measurement to maximize selection consistency across breeding cycles while maintaining the maximum amount of desired alleles among the candidates. Inclusion of the G × E effect in GS prediction models also proved to increase the consistency of selecting best performers across environments in order to accelerate the replicated-trial evaluation. When confidence on retaining superior lines while culling inferior ones can be achieved, higher selection pressure can be applied to only advance a smaller subset to be included in more multiple location trials; GS’s potential in optimizing the testing of DHs is therefore supported. Further, since the consistency of GS on grain yield can be acquired with confidence, lines comprising the intermediate portion of the distribution may also be selected for targeting on other desirable traits such as for high protein content and superior gluten quality for hard red winter wheat, to improve the return of investment of DH development. We expect the positive results obtained from this study, along with all these potential benefits could advocate the actual implementation of GS in wheat variety development.

## Electronic supplementary material

ESM 1 (DOCX 12 kb)
