# Supplementary material for: Practical application of genomic selection in a doubled-haploid winter wheat breeding program
Source: Mol Breed. 2017 Sep 3;37(10):117. doi: 10.1007/s11032-017-0715-8 (PMC5582076; doi:10.1007/s11032-017-0715-8)
Supplement: Supplementary file 1 — (DOCX 12 kb) [file 11032_2017_715_MOESM1_ESM.docx]

Supplementary Material

Table S1 Summary of infection assessment statistics (mean and standard deviation)

| Time point | Severity (RS) | | Incidence (RI) | |
| --- | --- | --- | --- | --- |
|  | Mean | SD | Mean | SD |
| May 5^th^ | 3.08 | 2.010 | 8.38 | 12.193 |
| May 11^th^ | 3.32 | 1.933 | 12.68 | 18.846 |

Table S2 Total number of SNPs in each subgroup

| SNP call rate threshold | | | | | | |
| --- | --- | --- | --- | --- | --- | --- |
|  | 0.25 | 0.4 | 0.5 | 0.6 | 0.75 |  |
| Total number of SNPs | 12,994 | 9,244 | 7,260 | 5,726 | 4,010 |  |

Fig. S1 Comparison of bandwidth parameter h based on predictive ability from RKHS (with year 2014 and 2015 as training population respectively) cross validation model (with SNP effect only) across marker missingness levels

Fig. S2 Comparison of bandwidth parameter h based on predictive ability from RKHS cross year prediction model (with year 2014 and 2015 as training population respectively) across different model components: G=only marker effect in the model; G+HD(cor)=marker effect in the model with heading date-corrected phenotype as response variable; G+HD(cov)=marker effect and heading date as covariate in the model; G+Rust(cov)=marker effect and disease index as covariate in the model; G+HD+Rust(cov)=marker effect and both heading date and disease index as covariates in the mode.
